# Efficacy of Pidotimod use in treating allergic rhinitis in a pediatric population

**DOI:** 10.1186/s13052-020-00859-8

**Published:** 2020-07-07

**Authors:** Giulia Brindisi, Anna Maria Zicari, Laura Schiavi, Alessandra Gori, Maria Pia Conte, Massimiliano Marazzato, Giovanna De Castro, Lucia Leonardi, Marzia Duse

**Affiliations:** 1 Department of Pediatrics, Policlinico Umberto I, Sapienza University, Rome, Italy; 2 Department of Public Health and Infectious Diseases, Microbiology Section, Sapienza University, Rome, Italy

**Keywords:** Pidotimod, Microbiota, Rhinomanometry, Nasal obstruction

## Abstract

**Background:**

Allergic rhinitis (AR) and adenoidal hypertrophy (AH) are the most frequent causative disorders of nasal obstruction in children, leading to recurrent respiratory infections. Both nasal cavities are colonized by a stable microbial community susceptible to environmental changes and *Staphylococcus aureus* seems to play the major role. Furthermore, nasal microbiota holds a large number and variety of viruses with upper respiratory tract infections. This local microbiota deserves attention because its modification could induce a virtuous cross-talking with the immune system, with a better clearance of pathogens. Although AR and AH present a different etiopathogenesis, they have in common a minimal chronic inflammation surrounding nasal obstruction; hence it would be challenging to evaluate the effect of an immunomodulator on this minimal chronic inflammation with possible clinical and microbiological effects. The aim of this study is therefore to evaluate the efficacy of an immunomoldulator (Pidotimod) on nasal obstruction in children with AR and/or AH and whether its action involves a variation of nasal microbiota.

**Methods:**

We enrolled 76 children: those with allergic rhinitis (AR) sensitized to dust mites entered the AR group, those with adenoidal hypertrophy (AH) the AH group, those with both conditions the AR/AH group and those without AR ± AH as controls (CTRL). At the first visit they performed: skin prick tests, nasal fiberoptic endoscopy, anterior rhinomanometry, nasal swabs. Children with.

AR ± AH started treatment with Pidotimod.

After 1 month they were re-evaluated performing the same procedures.

The primary outcome was the evaluation of nasal obstruction after treatment and the secondary outcome was the improvement of symptoms and the changes in nasal microflora.

**Results:**

All patients improved their mean nasal flow (mNF) in respect to the baseline. In AR children mNF reached that one of CTRL. In AH children±AR the mNF was lower in respect to CTRL and AR group. We did not find any differences among all the groups at the two different time points in nasal microflora.

**Conclusions:**

Pidotimod is able to give an improvement in nasal obstruction, especially in AR children but this effect seems to be not mediated by changes in nasal microbiota.

## Background

Chronic nasal obstruction is an underestimated condition that severely affects the quality of life of many patients, by impairing sleep quality. The resulting irritability and chronic fatigue reduce  cognitive function and school performance over time [1].

Allergic rhinitis (AR) and adenoidal hypertrophy (AH) are the most frequent causative disorders of nasal obstruction in children, leading to recurrent respiratory infections (RRI) and contributing to the development of rhinosinusitis and obstructive sleep apnea (OSA) [2].

The prevalence of AR has been estimated to be approximately 2 to 25% in children, depending on age. The chronic allergic inflammation of nasal mucosa is responsible for nasal discharge, itching, sneezing, blockage or severe congestion, but also for recurrent infections [3]. The peculiar cytokinic storm of allergic inflammation leads to an impairment of natural immunity with a reduced expression of Toll like receptors (TLRs) and a decrease of ɣInterferon (ɣ-IFN) production. This impairment is responsible for prolonged viral survival and increased viral replication, explaining why allergic children are more susceptible to Respiratory Syncytial Virus (RSV) and Rhinovirus (RV) infections [4]. Indeed, the target therapy of allergic diseases is allergy immunotherapy (AIT) with a desensitizing effect that not only causes improvement of allergic symptoms but also a significant reduction in RRI [5].

Also AH is very common in pre-school children, often associated with recurrent inflammation of upper airways and allergic diseases. Clinical features, due to nasal obstruction, include mouth breathing, snoring, hyponasal speech and adenoid facies. However AH is a dynamic and potentially reversible condition under medical therapy but chronic infections, either viral or bacterial, can keep the pad of adenoids enlarged for years [6].

How and if this susceptibility could be due to the microbiota of the nasal mucosa is still controversial. Nasal cavities are the less investigated ecosystem and only recently extensively studied, showing that are often colonized by a temporally stable microbial community susceptible to environmental changes [7].

Actinobacteria, mainly *Propionibacterium* and *Corynebacterium spp.*, and phylum of *Firmicutes*, in particular by the genus *Staphylococcus spp.*, are the predominant species. Among them, *Staphylococcus aureus* seems to play a major role, affecting the composition of the microbiota with its presence and proportion [8]. Furthermore, nasal microbiota holds a large number and variety of viruses, especially in preschool age children with upper respiratory tract infections (URTI). Susceptibility to URTI is para physiological in the first years of life and is due to the slow and incomplete maturation of the immune system.

Several strategies that involve the use of immunomodulators or pre-probiotics had been proposed, with encouraging results. The efficacy of immunomodulators could be due to the direct action on the mechanisms of natural and adaptive immunity [9]. As for probiotics, modifications of the microbiota due to the addition of selected bacteria could induce a virtuous cross-talking between the microbiota itself and the immune system, with a better clearance of pathogens.

Although AR and AH present a different etiopathogenesis, they have in common a minimal chronic inflammation surrounding nasal obstruction; hence it would be challenging to evaluate the effect of an immunomodulator on this minimal chronic inflammation with possible clinical and microbiological effects .

Therefore the aim of this study is to evaluate Pidotimod efficacy on nasal obstruction in children with AR and/or AH and whether its action involves a variation of nasal microbiota.

## Materials and methods

### Study population

We consecutively enrolled children between 6 and 12 years old referred as outpatients during the 2017Autumn season (September–November 2017). Children with nasal obstruction due to AR and sensitized to dust mites entered the AR-group; those with documented adenoid hypertrophy entered the AH-group or AR/AH group if both conditions had been diagnosed. Children without nasal obstruction nor sensitized are enrolled as CTRL with a final ratio of 1:4. Children with asthma, genetic diseases, cardiovascular and lung chronic diseases, craniofacial malformations, acute illness in progress and/or in the month preceding the study and allergic to allergens other than mites were ruled out of the study. The use of systemic and/or local steroids and antihistamines or any other therapy in the last 4 weeks, was considered as exclusion criteria.

Diagnosis of AR was formulated according to the Allergic Rhinitis and its Impact on Asthma (ARIA) criteria [10]. Diagnosis of AH was confirmed by an expert pediatric otorhinolaryngologist and its degree was defined using Cassano et al. criteria [11].

### Study design

At the first visit (T0), children performed a complete routine clinical exam, skin prick tests (SPTs), anterior rhinoscopy, functional evaluation with nasal fiberoptic endoscopy (NFE), active anterior rhinomanometry (AAR), spirometry and microbiological evaluation of the front nasal cavities with nasal swabs. In addition they filled out the nasal symptom score (NSS).

Children with nasal obstruction (AR, AH and AR/AH-group) started the treatment with Pidotimod (1 vial per day for 30 days) and after 1 month (T1) they were revalued with the same procedures as the enrollment.

The primary outcome of the study was to evaluate the variation of nasal obstruction measured as nasal function from baseline to 30 days after Pidotimod treatment.

The secondary outcomes were the detection of the improvement of nasal symptoms, through the patient’s compilation of the NSS and of any changes in the composition of the nasal microbial flora before and after Pidotimod treatment.

Written parental or guardian informed consent was obtained for all participants enrolled in this study. The study was approved by the Ethical Commitee of “Sapienza” University of Rome.

### Skin prick tests

SPTs were performed for *Dermatophagoides pteronyssinus*, *Dermatophagoides farinae*, cat, dog, birch pollen, grass pollen, alternaria and pellitory pollen (ALK Abell, Denmark), according to EAACI group [12]. The positive control solution was 10 mg/mL histamine hydrochloride and the negative control solution was glycerol-saline solution. Wheals ≥3 mm were considered positive.

### Active anterior rhinomanometry

Patients wore a face mask, close their mouth and breathed only with the nose in accordance with the International Committee on Standardization of Rhinomanometry [13]. In accordance with Zapletal et al., the degree of nasal obstruction, based on rhinomanometry test values, was estimated as fraction of predicted values (p.v.) of mean nasal flow (mNF): grade 1 corresponded to no obstruction (77–100% of p.v.); grade 2 to mild obstruction (66–76% of p.v.); grade 3 to moderate obstruction (55–65% of p.v.); grade 4 to severe obstruction (44–54% of p.v.) and grade 5 to very severe obstruction (less than 44% of p.v.) [14].

### Nasal Fiberoptic endoscopy

NFE was performed by an expert pediatric otorhinolaryngologist using a 2.7 mm diameter endoscope.

The degree of AH was assessed according to Cassano’s criteria: grade 1 corresponded to free choanal opening (< 25%); grade 2 to adenoids occluding the upper half of the choanal opening (50%) without tubarian ostium involvement; grade 3 to adenoids occluding 75% of the choanal opening, with partial Eustachian tube involvement; grade 4 to adenoids completely occluding the choanal opening associated with an unevaluable tubarian ostium [11].

### Spirometry

Spirometry was performed using the Quark PFT Ergo® device (Cosmed, Rome, Italy), in accordance with the American Thoracic Society (ATS) - European Respiratory Society (ERS) guidelines [15]. Before each test, the volume and flow were calibrated using a 3-l syringe.

The session was concluded after 3 technically acceptable maneuvers lasting no more than 15 min [16].

### Nasal swabs

Nasal samples were collected from nares of patients by a dry sterile swab. Each swab was rotated five times inside the nares and used for microbial isolation or for metagenomic analysis. The swabs were placed into the agar gel transport medium (Sterile transport swab, Oxoid) and sent to the microbiology laboratory for bacterial culture, isolation and recognition. Each swab was independently streaked on a set of culture media plates: blood agar, chocolate agar supplemented with or not with Bacitracin, Mannitol Salt Agar, MacConkey 3, Bile-esculin agar, Cetrimide, and.

Chromogenic Candida agar plates (all from Oxoid) and incubated for 24–48 h with or not 5% CO_2_ at 25 and 37 °C. Sabouraud dextrose agar plates were incubated up to 7 days. The number of bacteria present on swabs was quantified by counting the colonies grown on plates, and annotated as low (< 10^3^ CFU/plate), medium (10^3^–10^4^ CFU/plate) and heavy (> 10^4^–10^5^ CFU/plate). *Staphylococcus aureus* strains were identified by the presence of β-hemolysis on blood agar and by coagulase-positive reaction (Staphylase test, Oxoid) and finally by MALDITOF. All the bacteria isolated, were identified by MALDI-TOF, Matrix Assisted Laser Desorption Ionization Time-of-Flight.

### Nasal symptom score (NSS)

Children filled out the total NSS, a validated questionnaire assessing severity and frequency of runny nose, nasal congestion, sneezing and itchy nose. As for the severity and for frequency, the score assigned to each symptom was respectively: 0 = absent/never; 1 = mild, lightly annoying/once in a while; 2 = moderate, sometimes annoying/often; and 3 = severe, very annoying, always or almost of the times. The maximum total score was 24 [17].

### Statistical analysis

The presence of statistically significant differences between groups was evaluated by performing chi-square test for categorical data while Kruskal-Wallis H test followed by Dunn’s post-hoc test was used for continuous variables. Comparisons between groups at different time points were carried out by using Wilcoxon signed rank test. In all cases, a *p* value ≤0,05 was considered as statistically significant. For multiple comparisons, a Bonferroni corrected alpha value was considered to assess statistical significance.

## Results

Out of 76 children enrolled, a total of 70 complete the follow up and entered the study: 41 male (58.6%) and 29 female (41.4%) aging from 6 to 12 years (mean age 8.61 ± 1.87).

We reported 6 drop-out (between 9 and 12 years of age): 5 among controls and 1 in AR group, mostly due to a low family compliance; no any adverse event was recorded.

57 children presented nasal obstruction: 26 children with AR, 16 with AH and 15 with AR + AH. The remaining 13 children were considered as CTRL. No differences were observed concerning the distribution of age and sex between all the groups.

All the children enrolled (57 in treatment with Pidotimod and 13 controls) performed a second visit (T1) after 30 days (+/− 7 days).

### Active anterior rhinomanometry

**Fig. 1:**
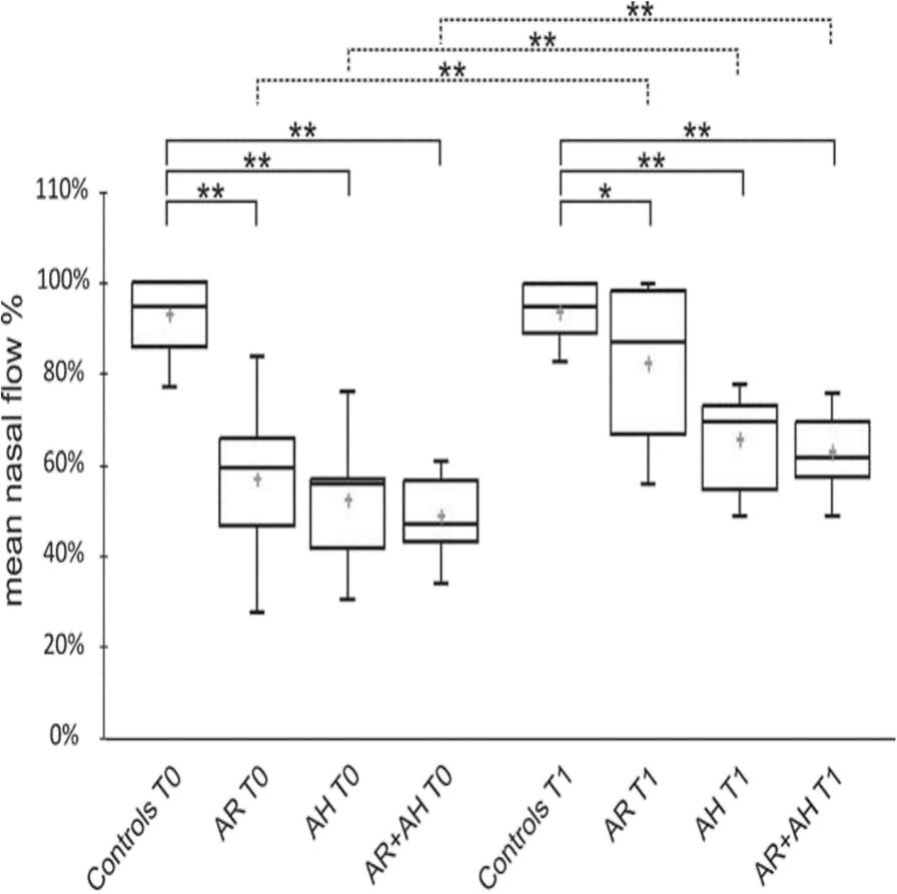
Box-Whisker plots showing mean, median and interquartile ranges of the % nasal flow in all the groups. Statistically significant differences between groups were reported for each separate time points (continuous lines) and between different time points (dotted lines). * *P* < 0.05, ** *P* < 0.001. AR: allergic rhinitis, AH: adenoidal hypertrophy

At first observation, nasal obstruction was moderate to severe in all the children and, as expected, patients in the AR/AH group showed more severe obstruction (mNF: 48.7% ± 9.12) in respect to those in the AH (mNF 52.2% ± 11.4) or in the AR group (mNF: 56.8% ± 14.2), although the difference between the groups were not statistically significant. Obviously, being a selection criteria, the nasal flow of CTRL was normal (mNF: 93.1% ± 8.02) and significantly higher than the one presented by the other groups (*p* = 0,000) (Fig. 1).

At the second visit (T1), after 30 days of Pidotimod treatment, all the children improved their mNF with a statistically significant increase in respect to the baseline value (*p* ≤ 0.001). The best response was observed among AR children where the mNF value (82.6% ± 15.8) approached that of CTRL (93.81% ± 5.94), while remaining significantly lower (*p* = 0.041). The intra-group comparison showed that the mNF of the AH group, regardless of the presence or absence of a concomitant AR (65.7% ± 9.9 and 62.9% ± 8.4, respectively), was not only significantly lower than the value of CTRL (*p* ≤ 0.000), but also that of one of the AR group (*p* = 0.001) (Fig. 1).

### Nasal symptom score (NSS)

About the NSS, the basal value score showed no statistical differences between disease groups and CTRL (AR 11.88 ± 4.46; AH 8.00 ± 4.56; AR + AH 11.73 ± 4.95; CTRL 8.46 ± 4.41).

At T1 we did not find any statistically significant variations (AR 10.54 ± 5.50; AH 9.50 ± 4.29;

AR + AH 11.20 ± 4.77; CRTL 6.18 ± 6.16).

**Fig. 2:**
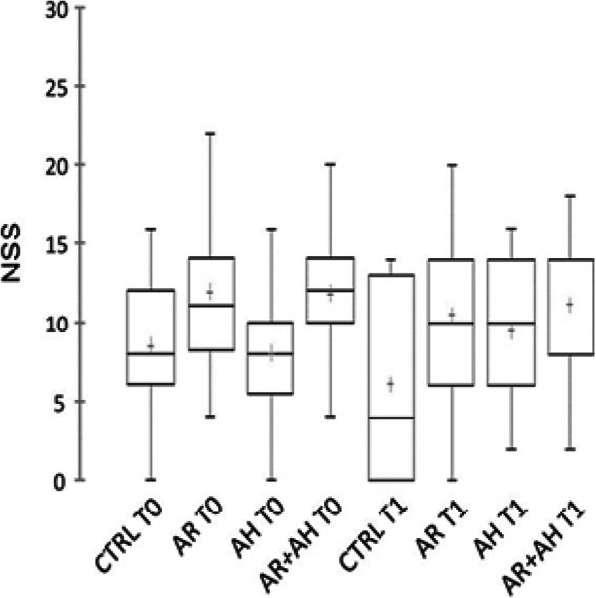
Box-Whisker plots showing mean, median and interquartile ranges of the nasal symptom score (NSS) in different groups of the diseased children and in controls. AR: allergic rhinitis, AH: adenoidal hypertrophy, CTRL: control group

Furthermore, the longitudinal analysis within the same group did not outline any differences in the NSS values before and after Pidotimod treatment. (Fig. 2).

### Spirometry

There are no significant differences in spirometric values both in the intra-group comparison both in the longitudinal analysis (data not shown). This data was expected because the presence of asthma was an exclusion criteria.

### Microbiology

**Fig. 3:**
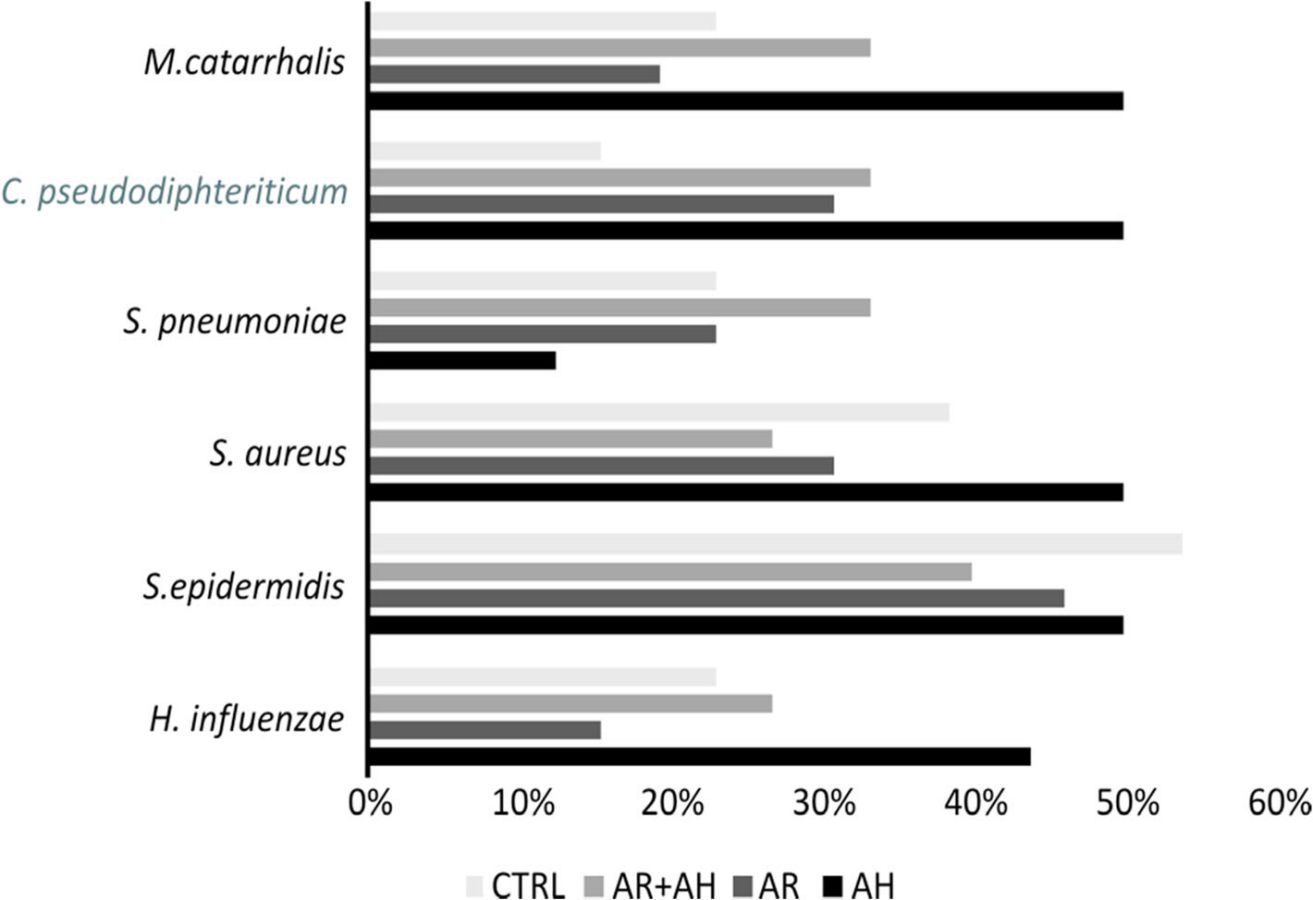
Bar plots showing the prevalence of bacterial species of clinical interest among different groups of studied subjects at first visit (T0) as determined by culture-dependent identification techniques. AR: allergic rhinitis, AH: adenoidal hypertrophy, CTRL: control group

**Fig. 4:**
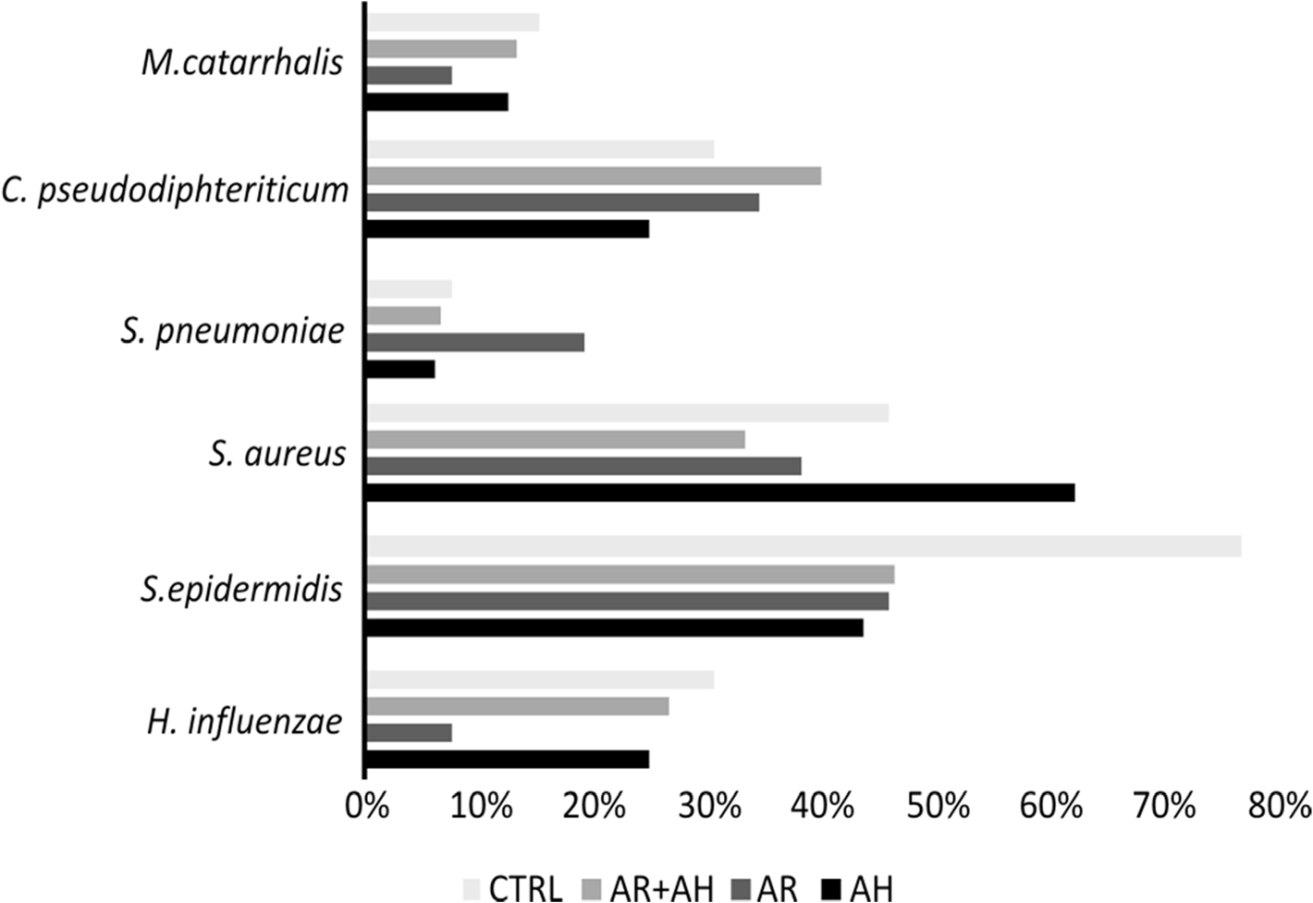
Bar plots showing the prevalence of bacterial species of clinical interest among different groups of studied subjects at second visit (T1) as determinedby culture-dependent identification techniques. AR: allergic rhinitis, AH: adenoidal hypertrophy, CTRL: control group

For what concerns the presence of microbial species, we analyzed the six most prevalent species in all the groups considered. In detail, we included *Moraxella catarrhalis*, *Corynebacterium pseudodiphteriticum*, *Streptococcus pneumoniae*, *Staphylococcus aureus*, *Streptococcus epidermidis* and *Haemophilus influentiae*. The results obtained showed the absence of differences in the prevalence of these bacteria among all the groups at the two different time points (Figs. 3 and 4).

## Discussion

This is the first study that evaluates the functional effects of Pidotimod on nasal obstruction in children with AR or AH.

Our data shows that the administration of Pidotimod is able to improve nasal flow significantly in few weeks and that this effect is not mediated by variations in the nasal microflora but it could be due to an inflammation decrease. The composition and prevalence of the species considered does not differ significantly either among children with AH, AR and AR + AH or CTRL.

In fact the microbiological profile of treated children and controls is not significantly changed after a month of Pidotimod therapy, confirming that nasal microbial population, at least in the front part of the nose, is relatively stable. The apparent prevalence of *Staphylococcus aureus* in children without nasal obstruction has no statistical relevance.

This data differs from recent observations suggesting that nasal microbial composition is linked to a specific clinical situation, such as AR [18].

However, it should be noted that results derived from various studies are not comparable because in the majority of them the target population consisted of adult subjects, with asthma or rhinosinusitis [19–21]. Moreover in all these studies, setting, case selection, sites and methods of sample collection were disparate. In fact, to date and to our knowledge, pediatric studies are relatively few and heterogeneous. Teo et al. studied microbioma of nasopharyngeal aspirates across the birth cohort of the CAS study, providing a characterization of bacterial communities within the human nasopharyngeal microbiome during the first year of life [22]. They found that early asymptomatic *Streptococcus* colonization at 2 months of age was significantly associated with chronic wheezing at 5 years of age, as consequence of a younger age of first lower respiratory infection (LRI). The antibiotic use disrupted asymptomatic colonization patterns leading to an increased risk of LRI and later asthma development. Our results, instead, refer to children of different ages, preschool and school age, where the microbiological profile has been strongly influenced by prolonged environmental stimuli. Given the physiological susceptibility to infections, it is not surprising that nasal microflora is heterogeneous with marginal differences between atopic and non atopic children at this age.

Similarly, Cardenas et al., identified a higher frequency of potential pathogens (Haemophilus, OR  =  2.12; *Staphylococcus spp*, OR  =  124) in oropharyngeal swabs samples of wheezy infants compared to controls [23]. Authors speculate that these bacteria might contribute to a wheezy diathesis in infants and to asthma later in life.

Since all children treated with Pidotimod demonstrate a measurable and significant reduction in nasal obstruction regardless of the underlying disease and without significant changes in the microbiota, this effect appears likely to be due to the direct action of Pidotimod on the immune response.

Pidotimod has shown an excellent profile of tolerability and safety in addition to a significant effectiveness in modulating immune response and in controlling RRI [24–26]. In vivo and in vitro studies showed that Pidotimod could improve immune response, affecting both adaptive both innate immunity. It is able to induce dendritic cells (DCs) maturation with the release of cytokines and other pro-inflammatory molecules, driving T-cells proliferation and differentiation towards a Th1 phenotype [27–29]. Furthermore, as shown in an ex-vivo study, Pidotimod could modulate the airway epithelial cells functions, up-regulating the expression of toll-like receptor-2 (TLR-2) on their surface which is known to recognize many bacteria, fungi and viruses [27].

Esposito et al., studying 20 children with community acquired pneumonia, demonstrated that Pidotimod administration in addition to standard antibiotic therapy might increase the natural immune response to an infectious stimulus, implementing maturation and function of the DCs [30]. We have indirectly observed an analogous rapid effect of Pidotimod in the analysis of intragroup differences in response to the therapy. After only 1 month, AR children have a significantly better nasal flow than AH children (with or without atopy) suggesting that this is due to its immunological action. Where in fact there is also an anatomical obstructive component, as in AH, the response is lower, because the resolution of the hypertrophy could be slower. However, it is possible that a specific antiallergic activity of the Pidotimod may also contribute to these results. In 2001 Gourgiotis et al. reported that Pidotimod induced in vitro a decrease in the expression of CD30 on peripheral blood mononuclear cells (PBMN) of atopic children [31]. More recently, Zhai et al. conducted a study on 60 children with asthma and AR treated with allergy immunotherapy (AIT), in which the study population was randomized for an additional treatment with Pidotimod. Children that received Pidotimod in addition to AIT, showed an improvement in the immunological parameters and in the respiratory function in respect to the controls. Therefore, it was supposed that Pidotimod was able to affect the Th1/Th2 balance and to have an anti-allergic role [32].

Our pilot study confirms that Pidotimod has a clinical and measurable effect on the stimulation of the immune system in children with inflammation and nasal obstruction, more evident in allergic subjects. The limits of our study consist above all in the number of cases and in the short time of observation. It is therefore conceivable that the prolongation of Pidotimod therapy in children with AH could lead to an obviously slow and progressive reduction. We did not clinically evaluate the intercurrent episodes and did not investigate the immunological studies.

However, we have considered nasal obstruction as a surrogate of an inflammatory condition and a susceptibility to RRI.

The strength of our study on one side is the choice of the sample population that allowed us to compare different types of inflammation and on the other side the study of parameters, such as the nasal flow, which is measurable and not operator-dependent.

In this prospective the discrepancy between objective results, such as the nasal flow, and the subjective ones like the NSS, confirm the importance of this detection. As we have previously shown, children underestimate the nasal obstruction and are unable to give a true measurement of their symptoms [33].

## Conclusion

This pilot study demonstrates for the first time that the use of an immunomodulatory molecule such as Pidotimod is able to give a rapid improvement on nasal obstruction, especially in AR children. This effect seems to be not mediated by consistent changes in nasal microbiota, so further studies are recommended to investigate the underlying immunological and microbiological mechanisms.

## Data Availability

All data analysed during this study are included in this published article.

## Abbreviations

AR: Allergic Rhinitis
AH: Adenoidal Hypertrophy
mNF: mean nasal flow
CTRL: Controls
RRI: Recurrent respiratory infections
OSA: Obstructive sleep apnea
ARIA: Allergic Rhinitis and its Impact on Asthma
RSV: Respiratory Syncytial Virus
RV: Rhinovirus
AIT: Allergy immunotherapy
SPT: Skin Prick Test
NFE: nasal fiberoptic endoscopy
AAR: Active anterior rhinomanometry
NSS: Nasal symptom score
URTI: Upper respiratory tract infections
